# Relational Disability and Invisible Illness in Industrial Britain

**DOI:** 10.1086/730431

**Published:** 2024-06-01

**Authors:** Coreen McGuire

## Abstract

Recent historiography has questioned the validity of an “industrialization thesis” that directly links disability to the industrial revolution and the exclusion of nonstandard bodies from factory work. In this article I defend the industrialization thesis by showing how an expanded version of it can reveal the disabling impacts of labor and illuminate the extent to which medical and legal standards diverged from lived experiences in the context of occupational disablement. Consideration of variance between compensated disability and experiential disability in conditions associated with disbelief, blame, and invisibility in both the historical and historiographical record highlights the value of using history of science approaches in epistemic considerations of disability in history.

Disability history and disability studies more broadly have thrived by exploring the insights of the social model of disability—a central tenet of disability studies. Crucially, this model differentiates impairment (of the individual body) from disablement (resulting from societal discrimination). Initially coined by activist and scholar Mike Oliver in 1983 at the same time as the Marxist group the Union of the Physically Impaired Against Segregation developed the concept, this binary model has been an incredibly powerful political tool. Oliver further reinforced the economic roots of disability in his classic work *The Politics of Disablement*. ^1^ Another founding member of the Union of the Physically Impaired Against Segregation, Vic Finkelstein, famously articulated how the capitalistic market forces of the industrial revolution gave “the machinery of production the decisive push which removed crippled people from social intercourse and transformed them into disabled people.”^2^ Such arguments linking the inability to work to the development of the modern category of disability are often held to be promoting “the industrialization thesis.” The industrialization thesis holds that capitalism is accountable for the use of disability as a social category, and the promotion of specific standards for the bodymind. The rise of industrial factory work standardized through Taylorism is highlighted as a major example of why nonstandard bodies that worked well in cooperative agrarian communities were increasingly excluded from the workplace.^3^ The explicitly Marxist commitment of this position has its origins in the development of the social model of disability and the successful development of this model in law continued this focus on materiality.

Historians Daniel Blackie and David Turner have urged us to question the simplicity of the industrialization thesis by using the example of coal mining to present evidence that, in fact, “bodily non-normativity *defined* workers in industrialising Britain.”^4^ Douglas Baynton’s oft-cited statement that “disability is everywhere in history” has been shown to be especially true in the British mining context thanks to the work of the Wellcome Trust–funded project “Disability and Industrial Society.”^5^ Thus, according to critics of the industrialization thesis, evidence of the ubiquity and acceptance of disability in industrializing Britain renders this theoretical position “empirically dubious.”^6^ Visible disability was normalized within mining communities.^7^ Moreover, the Disability and Industrial Society project has revealed that for most of industrial history, acquired disability was a collective experience.

Blackie develops a concise iteration of this criticism in his chapter in *The Oxford Handbook of Disability Histor*y, attacking the industrialization thesis on two fronts.^8^ First, he shows that disabled people worked throughout the industrial revolution; consequently, they were not excluded from the labor market in the way the thesis suggests. Second, he argues that the theoretical commitments of those promoting the industrialization thesis led them to overlook this evidence, thus casting doubt on the overall validity of the thesis. The argument that disabled people worked prior to and during the modern period in various industries is indisputable. However, this is not reason to discount the cogency of the industrialization thesis as a theoretical approach. The counter-argument that industrialization was not as wholly detrimental to the working lives of disabled people as has been assumed shows that industrialization was not detrimental to the lives of disabled people *only in that it did not preclude their ability to work*. This does not mean that modern work patterns were not detrimental to the lives of disabled people or that work was not disabling.

Understanding the relationship between labor and disability is crucial to our understanding of how disablement developed historically. For example, by linking disability and labor in the context of slavery, Stefanie Hunt-Kennedy reminds us that in a context in which people’s worth was “calculated based on their physical ability to perform hard labor,” disability and slavery were mutually reinforcing outside of the traditional chronology of the industrialization thesis.^9^ A more expansive definition of work reveals how identity categories intersect to affect disabled experiences. This conceptual expansion reveals how the relationship between disability and labor changes in different working contexts. Such ideas about disablement as an inevitable group experience linked to work is evident in the industrial Atlantic context, but as I argue in this chapter, this changed in twentieth-century Britain partly because of the development of welfare systems. As Sarah Rose has shown in the US context of railway workers, the mutual development of insurance and company liability shifted railway companies away from considering disabled workers as part of a group “with a culture that celebrated heroic self-sacrifice” to considering disablement to be a result of *individual* inefficiency and individual immorality.^10^ However, the racialized foundations of labor in the United States meant that the experience of twentieth-century labor was profoundly different for Black workers, whose access to compensation was controlled by insurance companies who set high premiums for Black lives on the basis that they posed greater health risks.^11^ Relatedly, Vicky Long and Victoria Brown emphasize the significance of workmen’s compensation to the development of theories that “apportioned blame to workers, rather than risky environments,” by stressing the individual’s genetic inferiority, innate susceptibility to accidents, or the personality traits common to women and lower-class workers.^12^

Such ideologies of individual immorality and inherent susceptibility to ill health were integrated into the development of the welfare state in twentieth-century Britain, which foretold intense interest in what Britain’s first chief medical officer called “the personal factor.”^13^ Increased state support augmented focus on an individual “health conscience” that involved adherence to the “maintenance of a high standard of personal health” necessary to prevent disabling individual bodies and thus the body of the state.^14^ The reconceptualization of health as an individual, mutable phenomenon rather than a shared experience entwined with the development of welfare and compensation cultures in the interwar era as “emphasising personal responsibility for health shifted liability.”^15^ This conceptual shift had important consequences in creating stigma around contested conditions in compensation disputes, thus associating those conditions with disbelief, blame, and invisibility in both the historical and the historiographical record.

For example, correcting for occupation in lung function measurements affected individual compensation because this practice led to repeated failures to identify pneumoconiosis from coal dust in mineworkers.^16^ Breathlessness that was not categorized or recognized by the medical systems meant that this kind of (often progressive) disability was not recorded in the same way that, for instance, accidents leading to amputations were. The same holds true for disability and illnesses that were fluctuating, progressive, actively concealed (potentially with the use of prostheses), stigmatized (such as psychological disability), or associated with malingering (such as miners’ nystagmus or fatigue). These kinds of experiences became known only in exceptional cases, and it is less clear that they were either accepted or normalized. Yet “low visibility” is not the same as “low incidence,” as Long and Brown have revealed through their work on mental illness and mining.^17^

In what follows, I assess low-visibility cases, beginning with a review of the compensation system in the British coal-mining industry in the first half of the twentieth century. I then use case studies of contested compensation claims to show how these rare examples hint at a hidden ecosystem of disability invisible in extant historiography. Twentieth-century measurement technologies led to significant divergences between the objective and subjective experiences of disability. I extend this thesis to legal frameworks of compensation and apply it to consider variance between compensated disability and experiential disability. This illuminates the ways in which medical and legal standards diverged from lived experiences in the context of occupational disablement. I argue that the case studies from the British coal-mining context are essential for illuminating the complexity of industrial disablement and call for a revision of the industrialization thesis.

Aligned with Rose’s argument that “the ‘problem’ of disability lay not in [workers’] actual impairments or the work they did but rather in the meanings attributed to those impairments by policy makers and employers,” I defend the (limited) utility of the industrialization thesis and maintain the importance of labor in defining and shaping standards of “normalcy” in twentieth-century Britain.^18^ This argument is not contingent on the British context, and equally works to affirm the significance of the industrialization thesis to the United States, especially during Reconstruction, when Black workers’ perceived inability to engage with twentieth-century industry shaped the “mutually rein-forcing categories” of race and disability.^19^

These contested disability experiences are doubly invisible in their absence from standardized compensation rubrics and subsequent erasure from the historical record and related historiography. Invisibility as a frame of analysis to explore invisible illness and hidden disability has been critiqued for the way it (ironically) occludes experiences that are not in fact invisible.^20^ I use *invisible disability* here to emphasize that although these experiences are not invisible to those experiencing them, they are unseen, unheard, or unacknowledged by those in power—often medical authorities, sometimes simply bureaucratic systems. In other words, they are invisible because they *are hidden* by these systems. Nonetheless, I make use of the concept of invisibility here to capture a range of diverse conditions that have few common features other than that they have been over-looked in the historiography of twentieth-century industrial disability.

Another reason the precise constitution of the categories used to define disability matters is their concrete policy consequences, which I explore in this article through detailed analysis of four illustrative case studies of claims for industrial disablement benefit put to the National Union of Mineworkers, Yorkshire Area, which were decided in 1951. The disablement benefit panel for coal mining formed part of the networks of pre–National Health Service care systems that had developed in the early twentieth century to moderate levels of compensation and care. They depended on a new binary conception of disability as either heritable (not compensable) or acquired (compensable). Establishing this binary shifted focus onto the individual, whose lifestyle and inheritance were now central to the measurement of their relative disability. This sorting process constructed a twentieth-century paradigm of *individual* health that depended on a concomitant reconceptualization of disability.

Moreover, I highlight that disability categorization depended upon other forms of body categorization. This work is intersectional, and considers how different social identities shaped different experiences of disability.^21^ Indeed, disability operates between and above categories such as race, gender, and class like a “meta-analytic umbrella” that covers how power operates among these intersections.^22^ For example, ideas about disability and biological differences between races affected twentieth-century British legislation, such as the various Aliens Acts that were mobilized to prevent immigrant groups entering Britain on the grounds of their disability.^23^ Disability was used to justify the exclusion of “unfit aliens” who belonged to “defective races” specifically on the grounds of heritable health.^24^

My methodological approach to this archival material draws upon the work of Ann Laura Stoler, who has traced the creation of social categories in Dutch colonial administration by “reading along the archival grain.” She writes that “working along the grain is not to follow a frictionless course but to enter a field of force and will to power.”^25^ Reading against the grain of the cases that motivate this article reveals the experiences of the disabled within the family, while reading *along* the grain of the archive exposes its taxonomic power. Approaching the archive as a classificatory force enables us to work out the decisions, issues, individuals, and structures that made “categorical claims about those social differences that mattered and those that did not.”^26^ This reveals the epistemological power of categorization in its creation of disability as a social difference that mattered and that could be counted, classified, and controlled.

## Invisibility In Medico-Legal Frameworks

The 1897 Workmen’s Compensation Act was the first piece of legislation to allow workers injured by occupational accidents in Britain to claim compensation from their employers, and in 1906 it was expanded to include industrial diseases as well as accidents.^27^ However, these diseases were limited to six specific conditions—anthrax, lead poisoning, mercury, phosphorus- and arsenic-related diseases, and ancylostomiasis. It excluded diseases due to dust.^28^ Amid the heady Liberal Reforms, this act was swiftly followed by the National Insurance Act of 1911, which created the first system of health insurance and sickness benefit for industrial workers in Britain.^29^

The same year, silicosis-specific compensation was introduced, but it explicitly excluded coal miners. This group would not be included under any legislation until 1929 with the advent of the Various Industries (Silicosis) Scheme of 1928, though this allowed for compensation only for total disability or death.^30^ Asbestosis followed in 1930. In 1931 a silicosis medical board was set up to moderate partial disability in miners, who could finally claim compensation thanks to the extension of the Various Industries Scheme. However, these schemes did not cover miners working underground until 1934, and compensation was granted only if the miner could prove exposure to silica dust. The scientific struggle to prove that a disease due to coal dust existed was undertaken by the Medical Research Council between 1942 and 1945, and it was duly recognized in compensation in 1943.^31^

There was thus a significant hierarchy of disability in the coal-mining compensation context, and the progressive nature of breathlessness augmented its invisibility. The long delay between the existence of respiratory illness due to coal dust and its acknowledgment affected how respiratory illness symptoms were experienced. This is especially pertinent to consider in view of occupational disablement, as “sensory meaning is never simply a question of physiology; it is always mediated by culture, in the sense of the ways of life, language, ritual practices, beliefs and aesthetics of a group, community or society.”^32^ Coal-mining communities of the twentieth century shared strong collective features and were notably united by a local culture, sense of place, and communal support networks.^33^ In the coal-mining context, the experience of group disablement meant that it was more difficult for miners with pneumoconiosis to recognize the subtle, embodied, individual nature of progressive breathlessness. As an autonomic bodily function, the experience of breathing often goes unseen and unheard unless it becomes pathological, and even then, its essential incommunicability is exacerbated by the poverty of narrative accounts to convey the experience.^34^ When breathlessness is wide-spread in the community, “local biologies make the experience of breathlessness socially inaudible.”^35^ The situation for coal miners for whom breathing was difficult was particularly complex. As Jane Macnaughton explains, “The inability to breathe silences people, and polluted air shuts those with breathing difficulties more firmly behind the protection of closed doors, unseen and ignored by society.”^36^

The process of compensating for respiratory illness in coal mining also hinged on categorization, as its measurement depended on specific classifications linked to race and class. The practice of “correcting for race” in spirometry, for instance, was refined as part of this process, as was correcting for occupation as a proxy for class.^37^ Lundy Braun has demonstrated how the use of categories in spirometry tests eventually essentialized racial differences to further obscure how social and environmental factors influence lung function.^38^ These classificatory practices were crucial in shaping the modern understanding of disability as an individual experience, and this development was paralleled in the history of compensation for byssinosis.

The existence of an industrial disease caused by inhaling cotton dust had been acknowledged in the nineteenth century, but it took until the twentieth century for health and safety legislation and compensation structures to develop. Byssinosis was a chronic and progressive respiratory illness caused by exposure to cotton dust, usually found in textile workers. The phrase “that Monday feeling” originated in workers with byssinosis who used it to describe the chest tightness and breathlessness they experienced when returning to work on a Monday. Byssinosis was not compensated until 1941 with the 1941 Byssinosis Benefit Scheme, but women (who formed most of this workforce) were entirely excluded from this compensation scheme. There was no reason given for this overt gendered discrimination. Legal historians Sue Bowden and Geoffrey Tweedale have suggested that it was simply because the industry was female dominated; thus, “the government excluded them to reduce costs, while targeting compensation at strippers and grinders, who were invariably male, unionized, and ‘vital’ to the industry.”^39^ The resulting invisibility of female claimants contributes to our understanding of how industrial disablement has been gendered as male, and why male industrial disablement has been prioritized in both labor history and disability history.^40^

Such compensation disputes focused on proving the influence of the individual over their environment while discounting the impact of the environment on the individual. This meant that an individual’s lifestyles and personal risk factors were elevated over the risks posed by their environment, as in the byssinosis court debates, which exploited “the environment/lifestyle debate concerning the way in which Lancastrian air pollution and the smoking habits of cotton workers contributed to their byssinosis.”^41^ Categorization was key to such arguments, and disability was key to the establishment of other normative social categories used in medicine and twentieth-century epidemiology. For instance, compensation amounts differed for women and men and differed between the married and unmarried.^42^ This is also clear from close analysis of the claims made under the 1952 Pneumoconiosis and Byssinosis Benefit Scheme, which forms the content of the following section. These examples connect through the evaluative focus on body competence, and the use of categorizations to make such evaluations.

In 1943 the Pneumoconiosis (Benefit) Scheme was enacted; following the end of the Second World War and the nationalization of the mines (in 1946), the scheme was replaced with the 1952 Pneumoconiosis and Byssinosis Benefit Scheme. The 1952 fund was designed to account for cases not covered by either the Workmen’s Compensation Acts or the Industrial Injuries Act and provided more workers with access to benefits. However, claimants were eligible only if they were deemed by both the benefit and the medical board to be totally and permanently disabled. Claimants had to have worked for “at least twenty years in one or more of the occupations covered for that disease,” and awards were for a maximum of forty shillings a week.^43^ The scheme accounted for a limited amount of funding for claimants’ wives and had a separate system of compensation for those who needed permanent assistance, but no compensation was specifically available for husbands of women claimants. Such gender distinctions remained relevant to the development of the unique terminology that developed to explain breathlessness as “that Monday feeling” in compensation contexts.

In a strange twist, the lay epidemiology that gave rise to the (mainly women) worker’s description of “that Monday feeling” was entirely subverted when compensation became more widely available in the 1970s and 1980s. Workers had to prove that they had a history of the “Monday feeling,” as diagnosis and compensation hinged on proving that they had had both the requisite Monday symptoms and the relevant occupational history. As Bowden and Tweedale explain: “This meant diagnostic and hence definitional problems, not least since many workers suffering from byssinosis did not consult a doctor until the disease was well developed—and hence presented with what appeared to be ordinary bronchitis. Demonstrating the requisite Monday symptoms and occupational history remained a problem (for the worker).”^44^ Michael Bloor has shown that the men working in coal mines mobilized scientific terms to lend credence to their own collective understanding of miner’s lung.^45^ Yet the women and men working in the cotton mills had the terminological tools they used to express their somatic experiences taken and used against them.

The National Health Service was formed in 1946 and replaced the insurance system in Britain. Consequently, the National Insurance (Industrial Injuries) Acts of 1946 and 1948 were designed to provide compensation rather than treatment. The 1952 scheme aimed to provide “payments out of the Industrial Injuries Fund for total disablement or death from pneumoconiosis or byssinosis in certain cases which are not covered by either the Workmen’s Compensation Acts or the Industrial Injuries Act.”^46^ Its mechanisms meant that the visibility of partial disability decreased again as it strictly stipulated, “If you are suffering from pneumoconiosis or byssinosis, but are not totally disabled, the Scheme does not apply to you; but if in the future your condition gets worse so that you become totally disabled by the disease, you can then make a claim.”^47^

Despite the widened scope of compensation legislation, there were disputed cases and rejected claimants. The system was bewildering and complicated and many cases fell outside of its scope or were rejected due to lack of evidence or bureaucratic contests. In such situations, the union would work to appeal the decision and in 1951 seven such cases for the Industrial Injuries Act were brought to the Industrial Injuries Commissioner by the Yorkshire area miner’s union.

## Invisibility and Hidden Disability

The first of these rejected cases was that of George Hames, whose case was debated at the Pontefract tribunal on April 9, 1951. Hames was a collier who was assessed at “20 per cent disablement” following a “right inguinal hernia.” However, it later became apparent that although Hames had returned to work and appeared able to do “full pre-accident work,” this illusion was only sustained through his use of a concealed truss and the collaboration of his colleagues and friends. The use of trusses was common for miners with hernias. This undergarment, or supportive pad, was attached to a belt designed to press on the hernia and thus keep it in place and relieve pain. The deputy commissioner explained that Hames could no longer use the pneumatic pick and was often behind in filling his coal on to the belt: “His workmates therefore told him ‘If you want to start, we will help you through.’ He could not use the pneumatic pick, nor set the timbers in his stint, and often was behind hand at the end of his shift in filling his coal on to the belt. All these are essential operations in a collier’s work, and he had to depend upon the goodwill and help of his mates working in the neighborhood who came and did the work for him.”^48^ This case reveals what Michael Worboys has recently described as a “symptom iceberg” consisting of the actions of “non-patients,” whose symptoms are managed by and within the community without professional involvement.^49^ This idea transfers elegantly to disability history, as Worboys acknowledges when he writes that “the management of chronic conditions, where the notion of the ‘post-patient’ might be useful, is *care* as much, if not more than, treatment.”^50^ It is additionally relevant to the idea of relational disability, and indeed “kinship ties” and “the significance of kinship to working life” have been highlighted by Blackie as particularly relevant to understanding of the experiences of disabled miners.^51^ This example illuminates how disability was managed by networks and relationships. Such discourses advance our understanding of disability as *relational*, and thus highlight how disability experiences were framed by family circumstances, including how disability affected family functioning in turn.^52^ Turner and Blackie have explored this interdependence in their exploration of “disablement as a collective working-class experience” by analyzing the relationships of an activist (Joseph Habergam) for whom “his brother and sister were more than mere living mobility aids … : they were allies bound together in a struggle for mutual survival.”^53^

British coal-mining culture was characterized by bonds of allyship that demanded caregiving, and as late as the 1950s encouraged “carrying” workers in this manner.^54^ Indeed, the commissioner used this terminology explicitly in his judgment when he wrote, “Unless his mates had been prepared to ‘carry’ him until the time of his operation (June, 1950) the claimant must have given up work. Although he continued with their help, he was not really capable of carrying out what had formerly been his regular occupation.”^55^ His mates working alongside him supported him not only in his work but also in his claim for an increase in the rate of his disablement pension, as it was the “joint statement signed by six of the claimant’s workmates” that provided the crucial evidence that eventually resulted in the successful increase of his disablement pension.

In this case and the following one, it is relevant to consider the actions of those who actively sought to disguise or conceal their disabling conditions. Hames “had to take shifts off because of his rupture,” and so was only able to continue working through pain management and by strategically resting. Although he “counts” as a worker, he was managing his pain in the home and by using various prostheses that helped him conceal his status as disabled. There was an additional imperative to conceal the impact of unusual or stigmatized conditions. Miner’s nystagmus, which the next case study is concerned with, is a condition in which the eyes oscillated and affected the miner’s vision. It was especially stigmatized due to the flexibility of its definition, its association with malingering, and its connection with psychoneuroses. Such rhetoric composes much of the content and case analysis contained within the Medical Research Council’s *Third Report of the Miner’s Nystagmus Committee*, published in 1932. Although this report initially links the condition with working in constant darkness, and even demonstrates a correlation between the use of electric lamps and a reduction in the condition’s frequency, its conclusions connect the condition to hysteria and malingering. For instance, the report states that “many conditions of neurosis are included owing to the loose definition of the disease.”^56^ It continued to argue that the condition was more likely to develop in “men already subject to psychoneurotic symptoms or tendencies,” and that “some may find in the eye troubles an escape from their other symptoms: for the substitution of mental symptoms for organic is reversible and primary mental symptoms may disappear with the onset of a hysteria with physical manifestations.”^57^ Such attribution of an “organic” disease to “functional” causes originated in the medical responses to shell shock and were also applied by psychiatrists to cases of apparent hysterical deafness, much to the chagrin of established otologists.^58^ Theories advanced after the First World War led to the idea that miners were either consciously malingering or unconsciously producing “symptoms as a means of withdrawing from a terrifying working environment.”^59^ These debates over causation worked to deny compensation by attributing compensable illness to individuals’ inherent “mental weaknesses.” However, the committee in the 1932 report was ambivalent about the question of whether such mental illness ought to be compensable, stating, “Whether there is a disability independent of any psychoneurotic element may be left an open question.”^60^

This question and the potential for psychological illness to be associated with malingering meant that trade unions emphasized the physical impacts on nystagmus and downplayed any associated mental health issues.^61^ James Bancroft’s claim before the tribunal in Doncaster on April 11, 1951, did not invite dispute about whether or not he had miner’s nystagmus but focused on whether the work he was now medically cleared to do was equivalent in terms of recompense to his mining work.^62^ The question was not whether he was disabled but rather whether the employment he was able to do was “of a standard equivalent to the employment that he followed in his regular occupation as a conveyer belt filler.”^63^ In this case, the definition of disablement was flexible, and distinctly social. The coal-mining context is crucial here, for as Turner and Blackie have noted, the intense physical labor associated with the most valuable coal-mining jobs skewed the standard of normalcy associated with miners’ bodies. The bodily standards expected of miners were generally considered to place them in a separate class, as George Orwell famously ruminated on in his descriptions of miners as “superhuman” in *The Road to Wigan Pier*. This vivid and vaguely homoerotic passage is worth quoting at length, as Orwell’s writing generally is:

It is only when you see miners down the mine and naked that you realize what splendid men, they are. Most of them are small (big men are at a disadvantage in that job) but nearly all of them have the most noble bodies; wide shoulders tapering to slender supple waists, and small pronounced buttocks and sinewy thighs, with not an ounce of waste flesh anywhere. In the hotter mines they wear only a pair of thin drawers, clogs and kneepads; in the hottest mines of all, only the clogs and knee-pads. You can hardly tell by the look of them whether they are young or old. They may be any age up to sixty or even sixty-five but when they are black and naked they all look alike.^64^

As I have previously explored, the notion that miners were a separate medical class fostered damaging ideologies within the measurement and assessment of pneumoconiosis. Because impairment and ill health in industrializing Britain was regarded as the “natural” consequence of work attendant to “a breed apart,” mining was regarded as a disabling group experience.^65^ Orwell’s emphasis on the blackness of the miners is further relevant because class shaped the body of the miner, but the resulting physical transformation was often viewed and described in racial terms. Racialized health frameworks scaffolded the gap that Stephanie Ward identifies between the “middle-class gaze and miners’ lived experience.”^66^

For claimant John Hammond, the medical officers’ assessment of his ability to work with pneumoconiosis diverged from his own doctors’, although the medical officer of the Ministry of Health admitted that “he was short of breath with exertion and coughed a lot and could not do work entailing severe exertion or exposure to damp, dust, or fumes.” Though the Ministry of Health continued to assert that the sixty-one-year-old could work as a watchman, the commissioner accepted the view of the claimant’s doctor and ruled, “The claimant could not, I think, reasonably be expected to work as a watchman, particularly in November, which is the period of the year with which I am concerned. I find great difficulty in thinking of anything at which he could work.”^67^ This judgment intimates that the commissioner considered the body to be the only suitable source of income for miners.

Contestation of illness was also central to the final case relevant to this article, which was the only case of the seven in these archives that was refused by both the insurance officer and the commissioner. In January 1949, Bertie fell down a flight of canteen steps (at work) in a way that caused serious injury to his testicles, leading to immediate medical shock and a longer period of incapacity, which developed into a “nervous” condition and eventually suicide. For Bertie’s wife, Florence, it was clear that the “balance of his mind was disturbed by reason of worry and ill-health” because of his injury and subsequent treatment.^68^ For the insurance officer and commissioner assessing her claim for industrial death benefit following the suicide, the “condition of the testicles had no causal relationship to the accident” and it so followed that “the psychological effect upon the deceased of the condition of his testicles can in no way be said to be attributable to the accident.”^69^

The kinds of ambiguities involved in the details of this case are characteristic of invisible illnesses, contested complaints, and stigmatized sickness. Such conditions collapse the distinctions between the able and the disabled that the compensation systems used by disablement benefit panels in the mid-twentieth century attempted to categorize and uphold. This was a particularly complex case because the Medical Board had initially upheld Bertie’s injury benefit claim. In addition, the coroner supported Florence’s claim and argued that “the deceased committed suicide while the balance of his mind was disturbed by reason of worry and ill-health including pain in the back from osteo-arthritis and disease of the testicles following injury at work some twelve months ago.”^70^

However, the pathologist who conducted the postmortem held the view that “the condition of the deceased’s testicles was of natural origin” and thus disputed whether the accident at work was linked to the testicle injury. By disputing this connection, the commissioner and insurance officer were able to deny Florence’s claim, casting doubt on the fact that Bertie had been disabled at work and suggesting that his condition was inherited or congenital. We see here again the shift to undermine occupational disability by stressing an individual’s natural disability. The causation debate worked to distract and obviate from the real issue at stake in this case, which was that the worry and pain that had resulted from the injury in the year in which he relied on disablement benefit presented a psychological trauma that contributed to his mental ill health and death. It also failed to consider the work-related osteoarthritis, and the psychological impact of living in constant pain. Long and Brown have demonstrated that witnessing mining accidents could result in “mental distress” that was compounded by the ways in which “the mechanisms of compensation led to the contestation of work-related mental distress.”^71^ This case reminds us of the need to focus on occupational disability in a community sense that goes beyond the working life of the individual. The idea of disability as relational is relevant to this case in several ways.

First, this case for industrial death benefit was to help Florence, Bertie’s wife. Women were caregivers in mining communities, and Florence’s role as a caregiver is pertinent. Women were often precluded from seeking work because of the need to care for husbands who had been injured in the mines.^72^ Second, caring for mineworkers was generally punishing and difficult, and took a hard toll on the women’s own bodies, which could then make it more difficult to seek work for themselves after their caregiving role finished. Alexandra Jones has argued that the impact of such general fatigue and chronic illness ought to be considered from a disability history perspective, despite the methodological problems it raises in relation to potential perpetuation of the medical model. Including such experiences expands our understandings of the way both gender and class intersected with disablement; thus, “just as our understanding of industrial work needs to be expanded, adopting a broad definition of disability is important to a consideration of coalfields women, whose disabilities were often related to chronic ill health, complications from childbirth and strain injuries from over-exertion.”^73^ They were excluded from the labor market because of disability, but not (necessarily or at least initially) their own. This creates methodological problems because there are no equivalent compensation records for disabled women, for, as Ward points out, “Women’s bodies were washed in private, medical complaints were often invisible, and the toil and exhaustion of domestic labour was rarely discussed.”^74^ Florence’s continued inability to work and need for this benefit may have resulted from her caregiving role. By taking a more expansive view of work to include caregiving and homemaking we can better understand the limitations of the traditional industrialization thesis and the potentials of using a moderate or expansive industrialization thesis. Reshaping the industrialization thesis to the new disability history offers exciting points of connections with innovative models of disability. The materialism that originally shaped the industrialization thesis can be fruitfully integrated with the post-social-model materialist approach articulated by Aimi Hamraie and the design model of disability formulated by Bess Williamson and Elizabeth Guffey.^75^

Third, the nature of coal mining as a community endeavor with strict norms of masculinity is relevant to our understanding of Bertie’s experience. Mining was a hypermasculine industry, in which the figure of “the big hewer” was elevated as a kind of superman. Ward has stressed the physical transformation of the body in coal mining by showing that the “relationship between muscles and masculinity within mining communities was enhanced by a wider cultural context which celebrated miners’ strength as a manly attribute.”^76^ Stopping work or even moving to less prestigious roles in the production process was associated with emasculation.^77^ These less prestigious roles were the ones originally done by women and children (before the Mines and Collieries Act of 1842 banned them), and recent work by Lucy Delap has shown that these roles were thereafter filled by intellectually disabled people who worked alongside those who became physically disabled during the course of their employment.^78^ The extent to which these roles were emasculating is ambiguous. Whether one was doing this role after becoming disabled in the process of work or whether one did this work as a congenitally disabled person played a critical role in the extent to which they were stigmatizing. What is less ambiguous is that work was associated with masculinity and the inability to provide fully for one’s family was both emasculating and stigmatized. Being unable to work in the mine changed the miner’s body in a way that was both alienating and distressing, as unemployment “disrupted the relationship between muscular bodies and the ideal masculine quality of physical strength.”^79^ As Julie Anderson has elucidated, focusing on the *interplay* between physical and mental trauma allows for further investigation of the mental trauma of injury and wounding, the connections between physical and mental trauma, and the emotional experiences of physical trauma.^80^ This is essential to grasp the balance of Bertie’s mind in the “period of incapacity” in which he was unable to work due to the injury to his testicles. Bertie’s case is also instructive in that it shows how differently stigmatized conditions can intersect and interact to devastating effect.

The injury to the testicles posed a direct and indirect threat to his manhood. This was a direct threat in that it suggested sexual trauma and failure (and inability to perform the sexual role of a husband) and an indirect threat in that the inability to work led to the inability to perform the role of the husband as provider. His testicle injury was doubly invisible and doubly stigmatized because of its connection with both sexual failure and occupational failure. Genital injuries were concealed both passively through clothes and actively through societal mores prohibiting certain kinds of discussion around their existence and use. Similarly, when soldiers received sexually related injuries in the First World War they were “largely ignored in public debates about returning servicemen.”^81^ Joanna Bourke has argued that “throughout the war and post-war years, it was accepted that a man’s penis and testicles were important signifiers of masculinity. Male sexuality placed inordinate emphasis on the active penis.”^82^ This context and loss added to the suffering and pain that Bertie suffered, compounded by the physical pain that attended his osteoarthritis. Chronic pain is invisible, stigmatized, and subject to disbelief. The experience of pain is also awkwardly situated in disability history historiography because those in pain may find it harder to accept or take pride in being disabled as pain poses a high cost to quality of life (unlike the cases of most disabled people, who report higher-than-average quality of life).^83^ In addition, the need and desire for diagnosis, cure, and medical intervention is at odds with a (strong) social model approach.

Finally, it is clear from the final decision made on this case that mental illness was not considered to be a relevant injury, as the disturbance to the balance of Bertie’s mind was neither denied nor affirmed, but simply ignored. Even though the original appeal to the local tribunal was satisfied that “the accident in January, 1949, caused the shock and nervous condition which led to the deceased taking his life,” this was dismissed because of the disputed causation linking the accident to the testicle condition. Acknowledgment of the high incidence of occupational mental illness in mining was hidden partly through compensation practices.^84^ Long and Brown have shown that compensation processes actively worked to repress “experiences of work-related mental distress” and deprioritized work-related mental disturbance by elevating physical health.^85^ Psychological injury was not taken as seriously as the injury for which there was physical evidence. It was invisible in both a diagnostic and a social sense; the cost of disclosing mental illness was high in 1951. Suicide remained a criminal act in England and Wales until 1961. This invisibility persists in the historiography of industrial disablement, which has “focused on the risks posed by work to physical as opposed to mental health.”^86^

## Conclusion

Further research could fruitfully explore the relationships and intersections between disability history focused on labor and existing medical history focused on stress and nervous conditions. An expanded industrialization thesis could offer ways to explore the ways in which “mind work” affected mental illness in groups more likely to be considered as middle class. These gaps can be explained both by the Marxist origins of disability history and the uneasy relationship between disability history and the history of psychiatry. This is largely a matter of perspective; perspectives can be shifted. In Michael Rembis, Catherine Kudlick, and Kim E. Nielsen’s words: “Where one historian sees a patient, another sees an inmate; where one historian sees a desire to treat and relieve suffering, another sees the desire to control socially deviant behavior; where one historian sees rehabilitation and occupational therapy, another sees the exploitation of a captive labor force; where one historian may focus on violent and abusive patients, another may focus on violent and abusive attendants and sadistic physicians; where one historian sees signs or symptoms of illness or pathology, another sees acts of resistance.”^87^ The possibilities of interdisciplinary work engaged with neurodiversity approaches and mad studies offer exciting possibilities for future work at this intersection. More pertinently for this collection, I conclude that applying history of science approaches to compensable disability allows us to question the very nature of disability. The idea that categorization systems do particular epistemological work is inherent to the work of historians of science.^88^ By asking, “What social groups are classified, corralled, coerced, and capitalized upon so others are free to tinker, experiment, design and engineer the future?” Ruha Benjamin has shown how this process entrenches race categories in technoscience.^89^ A history of science approach that considers the power of categories opens up new ways for disability historians to excavate hidden experiences by problematizing the categories themselves, emphasizing their epistemic authority and their imaginative power, and—most importantly—highlighting disabled resistance to them.

## Footnotes

1 Michael Oliver and Colin Barnes, *The New Politics of Disablement* (Basingstoke, UK: Palgrave Macmillan, 2012).

2 Vic Finkelstein, “Disability and the Helper/Helped Relationship: An Historical View,” first published in *Handicap in a Social World*, ed. Ann Brechin, Penny Liddiard, and John Swain (London: Hodder and Stoughton, 1981), now available as a PDF at https://disability-studies.leeds.ac.uk/wp-content/uploads/sites/40/library/finkelstein-Helper-Helped-Relationship.pdf, p. 3.

3 Penny L. Richards, “Industrialization,” in *Encyclopedia of American Disability History*, ed. Susan Burch, vol. 2: F–P (New York: Facts on File, 2009), 482.

4 David M. Turner and Daniel Blackie, *Disability in the Industrial Revolution: Physical Impairment in British Coalmining, 1780–1880* (Manchester: Manchester Univ. Press, 2018), 7; emphasis in the original.

5 Douglas C. Baynton, “Slaves, Immigrants, and Suffragists: The Uses of Disability in Citizenship Debates,” *PMLA* 120, no. 2 (2005): 562–67. For more information about the Disability and Industrial Society project, see their website, https://www.dis-ind-soc.org.uk/.

6 Daniel Blackie, “Disability and Work during the Industrial Revolution in Britain,” in *The Oxford Handbook of Disability History*, ed. Michael Rembis, Catherine Kudlick, and Kim E. Nielsen (Oxford: Oxford Univ. Press), 185.

7 Kirsti Bohata et al., *Disability in Industrial Britain: A Cultural and Literary History of Impairment in the Coal Industry, 1880–1948* (Manchester: Manchester Univ. Press, 2020).

8 Blackie, “Disability and Work during the Industrial Revolution.”

9 Stefanie Hunt-Kennedy, *Between Fitness and Death: Disability and Slavery in the Caribbean* (Urbana: Univ. of Illinois Press, 2020), 72.

10 Sarah F. Rose, *No Right to be Idle: The Invention of Disability, 1840sȓ1930s* (Chapel Hill: Univ. of North Carolina Press, 2017), 124.

11 Dan Bouk, *How Our Days Became Numbered: Risk and the Rise of the Statistical Individual* (Chicago: Univ. of Chicago Press, 2015).

12 Vicky Long and Victoria Brown, “Conceptualizing Work-Related Mental Distress in the British Coalfields (c.1900–1950),” *Palgrave Communications* 4, no. 133 (2018): 3.

13 Ina Zweinger-Bargielowska, *Managing the Body: Beauty, Health, and Fitness in Britain, 1880–1939* (Oxford: Oxford Univ. Press, 2010), 86.

14 Zweinger-Bargielowska, *Managing the Body*, 86.

15 Alex Mold et al., *Placing the Public in Public Health in Post-war Britain, 1948–2012* (London: Palgrave Macmillan, 2019), 36.

16 Coreen McGuire, “‘X-Rays Don’t Tell Lies’: The Medical Research Council and the Measurement of Respiratory Disability, 1936–1945,” *British Journal for the History of Science* 52, no. 3 (2019): 447–65.

17 Long and Brown, “Conceptualizing Work-Related Mental Distress,” 2.

18 Rose, *No Right to be Idle*, 3.

19 Paul Lawrie, “Race, Work, and Disability in Progressive Era United States,” in Rembis, Kudlick, and Nielsen, *Oxford Handbook of Disability History*, 229.

20 Alice Malpass, Coreen McGuire, and Jane Macnaughton, “‘The Body Says It’: The Difficulty of Measuring and Communicating Sensations of Breathlessness,” *Medical Humanities* 48, no. 1 (2022): 63–75.

21 Kimberle Crenshaw, “Mapping the Margins: Intersectionality, Identity Politics, and Violence against Women of Color,” *Stanford Law Review* 43, no. 6 (1991): 1241–99.

22 Beth Linker and Emily K. Abel, “Integrating Disability, Transforming Disease History: Tuberculosis and Its Past,” in *Civil Disabilities: Citizenship, Membership, and Belonging*, ed. Nancy J. Hirschmann and Beth Linker (Philadelphia: Univ. of Pennsylvania Press, 2014), 84.

23 British Nationality and Status of Aliens Act 1914, 4 Geo. 5, c. 17, s. 5(3), and s. 27(1), London, His Majesty’s Stationery Office.

24 See 1926 British Eugenics Society Pamphlet *Host or Doormat*, pp. 11–17, in “Immigration and Emigration” (1925–1958), reference no. SA/EUG/D.103, Eugenics Society Archive, London. For work on this in the US context see Baynton, “Slaves, Immigrants, and Suffragists,” and Douglas C. Baynton, *Defectives in the Land: Disability and Immigration in the Age of Eugenics* (Chicago: Univ. of Chicago Press, 2016). For work on links between disease (not disability) and immigration in the UK context, see Krista Maglen, “Importing Trachoma: The Introduction into Britain of American Ideas of an ‘Immigrant Disease,’ 1892–1906,” *Historical Studies in Ethnicity, Migration and Diaspora* 23, no. 1 (2005): 80–99.

25 Ann Laura Stoler, *Along the Archival Grain: Epistemic Anxieties and Colonial Common Sense* (Princeton, NJ: Princeton Univ. Press, 2008), 53.

26 Stoler, *Along the Archival Grain*, 32.

27 However, as Hunt-Kennedy has shown, this legislation was preceded by compensation legislation for British people in the Caribbean who were injured while chasing runaway slaves. Hunt-Kennedy, *Between Fitness and Death*.

28 Sue Bowden and Geoffrey Tweedale, “Poisoned by the Fluff: Compensation and Litigation for Byssinosis in the Lancashire Cotton Industry,” *Journal of Law and Society* 29, no. 4 (2002): 563.

29 Pat Thane, *The Foundations of the Welfare State*, 2nd ed. (Abingdon, UK: Routledge, 1996), 79.

30 McGuire, “X-Rays Don’t Tell Lies,” 457.

31 For a comprehensive overview of these developments see McGuire, “X-Rays Don’t Tell Lies,” 460.

32 Malpass, McGuire, and Macnaughton, “Body Says It,” 71.

33 For instance, Ben Curtis and Steven Thompson have explored the welfare systems set up by Welsh miners’ unions. See “‘A Plentiful Crop of Cripples Made by All This Progress’: Disability, Artificial Limbs and Working-Class Mutualism in the South Wales Coalfield, 1890–1948,” *Social History of Medicine* 27, no. 4 (2014): 708–27.

34 Havi Carel, *Phenomenology of Illness* (Oxford: Oxford Univ. Press, 2016), 109–10.

35 Malpass, McGuire, and Macnaughton, “Body Says It,” 71.

36 Jane Macnaughton, “Making Breath Visible: Reflections on Relations between Bodies, Breath and World in the Critical Medical Humanities,” *Body and Society* 26, no. 2 (2020): 31.

37 Lundy Braun, *Breathing Race into the Machine: The Surprising Career of the Spirometer from Plantation to Genetics* (Minneapolis: Univ. of Minnesota Press, 2014); Coreen McGuire, *Measuring Difference, Numbering Normal: Setting the Standards for Disability in the Interwar Period* (Manchester: Manchester Univ. Press, 2020).

38 Braun, *Breathing Race into the Machine*.

39 Bowden and Tweedale, “Poisoned by the Fluff,” 565.

40 David M. Turner and Daniel Blackie, “Disability and Political Activism in Industrialising Britain, c. 1830–1850,” *Social History* 47, no. 2 (2022): 128.

41 Bowden and Tweedale, “Poisoned by the Fluff,” 573.

42 *Departmental Committee on Workmen’s Compensation, Report to the Right Honourable The Secretary of State for the Home Department* (London: His Majesty’s Stationery Office, 1920), Section (a) Total Incapacity, 42.

43 Pneumoconiosis and Byssinosis Benefit Scheme, Mining Printed Ephemera re Damages and Injuries from the NUM (Yorkshire Area), 1952, accession number Misc. 2017/18:25, Special Collections, Durham University.

44 Bowden and Tweedale, “Poisoned by the Fluff,” 565–66.

45 Michael Bloor, “The South Wales Miners Federation, Miners’ Lung and the Instrumental Use of Expertise, 1900–1950,” *Social Studies of Science* 30, no. 1 (2000): 125–40.

46 Outline of Schemes for Supplementing Certain Payments under the Industrial Injuries Act, the Workmen’s Compensation Acts and the Pneumoconiosis (Benefit) Scheme (London, August 1948), in Mining Printed Ephemera re Damages and Injuries from the NUM (Yorkshire Area), accession number misc. 2017/18:25, Special Collections, Durham University (hereafter cited as Outline of Schemes, 1948).

47 Outline of Schemes, 1948.

48 National Union of Mineworkers, Yorkshire Area, Miners’ Offices, Barnsley, Claims for Industrial Disablement Benefit, Decision of the Commissioner case no. 35/2, June 13, 1951, Mining Printed Ephemera re Damages and Injuries from the NUM (Yorkshire Area), accession number Misc. 2017/18:25, Special Collections, Durham University.

49 Michael Worboys, “The Non-patient’s View,” in *Patient Voices in Britain, 1840–1948*, ed. Anne Hanley and Jessica Meyer (Manchester: Manchester Univ. Press, 2021), 33–60.

50 Worboys, “Non-patient’s View,” 54; emphasis mine.

51 Blackie, “Disability and Work during the Industrial Revolution,” 187.

52 Ben Curtis and Steven Thompson, “Disability and the Family in South Wales Coalfield Society, c. 1920–1939,” *Family and Community History* 20, no. 1 (2017): 25–44.

53 Turner and Blackie, “Disability and Political Activism,” 130.

54 Indeed, this case is from 1951 and carrying it seems to be accepted and normalized. Blackie, “Disability and Work during the Industrial Revolution,” 14.

55 National Union of Mineworkers, Yorkshire Area, Miners’ Offices, Barnsley, Claims for Industrial Disablement Benefit, Decision of the Commissioner case no. 35/2, June 13, 1951.

56 Medical Research Council, *Third Report of the Miners’ Nystagmus Committee* (London: His Majesty’s Stationery Office, 1932), 24–25, reference no. FD4/ 176, National Archives, London.

57 Medical Research Council, *Third Report of the Miners’ Nystagmus Committee*.

58 McGuire, *Measuring Difference, Numbering Normal*.

59 Long and Brown, “Conceptualizing Work-Related Mental Distress,” 7.

60 Medical Research Council, *Third Report of the Miners’ Nystagmus Committee*.

61 Long and Brown, “Conceptualizing Work-Related Mental Distress,” 7.

62 National Union of Mineworkers, Yorkshire Area, Miners’ Offices, Barnsley, Claims for Industrial Disablement Benefit, Decision of the Commissioner case no. 35/2, June 13, 1951.

63 National Union of Mineworkers, Yorkshire Area, Miners’ Offices, Barnsley, Claims for Industrial Disablement Benefit, Decision of the Commissioner case no. 35/2, June 13, 1951.

64 George Orwell, *The Road to Wigan Pier* (1932; repr., India: Prabhat Books, 2017), 18.

65 Blackie, “Disability and Work during the Industrial Revolution,” 7.

66 Stephanie Ward, “Miners’ Bodies and Masculine Identity in Britain, c.1900–1950,” *Cultural and Social History* 18, no. 3 (2021): 446.

67 National Union of Mineworkers, Yorkshire Area, Miners’ Offices, Barnsley, Claims for Industrial Disablement Benefit, Decision of the Commissioner case no. 35/2, June 13, 1951.

68 National Union of Mineworkers, Yorkshire Area, Miners’ Offices, Barnsley, Claims for Industrial Disablement Benefit, Decision of the Commissioner case no. 35/2, June 13, 1951.

69 National Union of Mineworkers, Yorkshire Area, Miners’ Offices, Barnsley, Claims for Industrial Disablement Benefit, Decision of the Commissioner case no. 35/2, June 13, 1951.

70 National Union of Mineworkers, Yorkshire Area, Miners’ Offices, Barnsley, Claims for Industrial Disablement Benefit, Decision of the Commissioner case no. 35/2, June 13, 1951.

71 Long and Brown, “Conceptualizing Work-Related Mental Distress,” 6.

72 Bohata et al., *Disability in Industrial Britain*.

73 Alexandra Jones, “‘Her Body [Was] like a Hard-Worked Machine’: Women’s Work and Disability in Coalfields Literature, 1880–1950,” *Disability Studies Quarterly* 37, no. 4 (2017), https://doi.org/10.18061/dsq.v37i4.6103.

74 Ward, “Miners’ Bodies and Masculine Identity in Britain,” 456.

75 Bess Williamson and Elizabeth Guffey, eds., *Making Disability Modern: Design Histories* (London: Bloomsbury, 2020); and Aimi Hamraie, *Building Access: Universal Design and the Politics of Disability* (Minnesota: Univ. of Minnesota Press, 2017).

76 Ward, “Miners’ Bodies and Masculine Identity in Britain,” 448.

77 Turner and Blackie, *Disability in the Industrial Revolution*.

78 Lucy Delap, “‘She Has Plenty of Go in Him’: Intersections of Gender and Disability in Twentieth-Century Britain,” keynote lecture for the Women’s History Network, March 8, 2020.

79 Ward, “Miners’ Bodies and Masculine Identity in Britain,” 450.

80 The emotional experience of physical trauma could present in further physical symptoms, as in the exploration of the psychosomatic condition “jumpy stump” that Julie Anderson explores. See Anderson, “‘Jumpy Stump’: Amputation and Trauma in the First World War,” *First World War Studies* 6, no. 1 (2015): 9–19.

81 Joanna Bourke, “Love and Limblessness: Male Heterosexuality, Disability, and the Great War,” *Journal of War and Culture Studies* 9, no. 1 (2016): 7.

82 Bourke, “Love and Limblessness,” 6.

83 See Havi Carel, “Ill, but Well: A Phenomenology of Well-Being in Chronic Illness,” in *Disability and the Good Human Life*, ed. Jerome E. Bickenbach, Franziska Felder, and Barbara Schmitz (Cambridge: Cambridge Univ. Press, 2014), 243–70; Gary L. Albrecht and Patrick J. Devlieger, “The Disability Paradox: High Quality of Life against All Odds,” *Social Science and Medicine* 48, no. 8 (1999): 977–88.

84 The idea that stress was related to certain kinds of work was acknowledged and this is an important intersecting literature. Mark Jackson, *The Age of Stress: Science and the Search for Stability* (Oxford: Oxford Univ. Press, 2013); Amelia Bonea et al., *Anxious Times: Medicine and Modernity in Nineteenth-Century Britain* (Pittsburgh: Univ. of Pittsburgh Press, 2019).

85 Long and Brown, “Conceptualizing Work-Related Mental Distress,” 2.

86 Long and Brown, “Conceptualizing Work-Related Mental Distress,” 2.

87 Michael Rembis, Catherine Kudlick, and Kim E. Nielsen, introduction to Rembis, Kudlick, and Nielsen, *Oxford Handbook of Disability History*, 9.

88 Geoffrey C. Bowker and Susan Leigh Star, *Sorting Things Out: Classification and Its Consequences* (Cambridge: MIT Press, 2000).

89 Ruha Benjamin, *Captivating Technology: Race, Carceral Technoscience and Liberatory Imagination in Everyday Life* (Durham, NC: Duke Univ. Press, 2019), 4.

